# Dataset for Phase I randomized clinical trial for safety and tolerability of GET 73 in single and repeated ascending doses including preliminary pharmacokinetic parameters

**DOI:** 10.1016/j.dib.2017.09.018

**Published:** 2017-09-17

**Authors:** Carolina L. Haass-Koffler, Kimberly Goodyear, Victoria M. Long, Harrison H. Tran, Antonella Loche, Roberto Cacciaglia, Robert M. Swift, Lorenzo Leggio

**Affiliations:** a Center for Alcohol and Addiction Studies, Department of Psychiatry and Human Behavior, Brown University, Providence, RI, USA; b Center for Alcohol and Addiction Studies, Department of Behavioral and Social Sciences, Brown University, Providence, RI, USA; c Laboratorio CT, San Remo, Italy

**Keywords:** Glutamate receptor subtype 5 (mGlu5), Allosteric modulator, GET 73, Safety, Tolerability

## Abstract

The data in this article outline the methods used for the administration of GET 73 in the first time-in-human manuscript entitled “Phase I randomized clinical trial for the safety, tolerability and preliminary pharmacokinetics of the mGluR5 negative allosteric modulator GET 73 following single and repeated doses in healthy male volunteers” (Haass-Koffler et al., 2017) [1]. Data sets are provided in two different manners. The first series of tables provided includes procedural information about the experiments conducted. The next series of tables provided includes Pharmacokinetic (PK) parameters for GET 73 and its main metabolite MET 2. This set of data is comprised by two experiments: *Experiment 1* references a single ascending dose administration of GET 73 and *Experiment 2* references a repeated ascending dose administration of GET 73.

**Specifications Table**

**Table:** 

Subject area	Pharmaceutical Sciences
More specific subject area	Phase I randomized clinical trial, first-time-in-humans, dose escalation
Type of data	Table
How data was acquired	Clinical assessments and blood sampling
Data format	Filtered
Experimental factors	The trial was approved by the Medicine and Healthcare products
	Regulatory Agency (MHRA). Participants (*N* = 80, total across the two experiments) are administered GET 73 either as single escalation dose [10, 30, 100, 300, 450, or 600-mg) or a repeated dose (100, 300, 450, 450-mg (twice a day)] over 14 days
Experimental features	This was a double-blind, placebo-controlled, ascending dose, Phase I randomized clinical trial conducted in healthy male volunteers in two Experiments. The primary aim was to look at the safety and tolerability of GET 73. In addition, preliminary pharmacokinetic data on GET 73 and its main metabolite MET 2 were collected
Data source location	LCG Bioscience, Cambridge, UK
Data accessibility	The data are available in this article

**Value of the data**

• Data provides safe doses for administration of GET 73.
• Data outlines the procedures tested for safe administration of GET 73 at safe doses in future study groups.
• Pharmacokinetic parameters were collected for GET 73 and MET 2 as reference data to evaluate the bioavailability of GET 73.

## 1 Data

The dataset presents an outline of the study and pharmacokinetic analytical data for GET 73: (N-[4-(trifluoromethyl)benzyl]-4-methoxybutyramide) (Haass-Koffler et al., 2017) [1]. Pharmacokinetic data for MET 2: (4-oxo-4-{[(4-trifluoromethyl)benzyl]amino}butanoic acid), a main metabolite of GET 73, is also included in the data set. Table 1, Table 2, Table 3, Table 4, Table 5 present procedural outlines for the Phase I randomized clinical trial. Table 6, Table 7, Table 8, Table 9, Table 10 provide pharmacokinetic data for GET 73, while Table 11, Table 12, Table 13, Table 14 provide pharmacokinetic data for MET 2.

**Table 1:** *Experiment 1,* single ascending dose schedule.

**Phase**	**Screening**	**Inpatient period**	**Study drug administration**	**Outpatient visit**	**Follow-up**
**Days**	−21 to −2	−1 to 3	1	3	15

**Table 2:** *Experiment 2,* multiple ascending dose schedule.

**Phase**	**Screening**	**Inpatient period**	**Study drug administration**	**Outpatient visits**	**Residency period**	**Follow-up**
**Days**	−21 to −2	−1 to 3	1 to 14	5, 7, 9, 11, and 13	13 to 16	28

**Table 3:** *Experiment 1,* assessments and procedures.

**Table 4:** *Experiment 2,* assessments and procedures.

**Table 5:** Clinical laboratory assessments.

**Clinical chemistry**	**Haematology**
γ Glutamyl Transferase	Haematocrit
Alanine Transaminase	Haemoglobin
Aspartate Transaminase	
Alkaline Phosphatase	Mean Corpuscular Haemoglobin
Potassium	
Sodium	Mean Corpuscular Hemoglobin Concentration
Calcium	Mean Cell Volume
Bilirubin – Direct (only if Total Bilirubin was outside the normal range)	Platelet count
Bilirubin – Total	Red Blood Cell count
Albumin	Urinalysis
Protein – Total	pH
White Blood Cell count (1)	Leukocytes (2)
Creatinine	Nitrite
Glucose (fasting)	Glucose
Inorganic Phosphate	Ketones
Triglycerides	Protein (2)
Cholesterol	Blood (2)
Urea	Urine test for drugs of abuse
Uric Acid	AmphetaminesEcstasy
Serum virology	Barbiturates
Hepatitis B Core Antibody	Benzodiazepines
Hepatitis B Surface Antigen	Cannabis
Hepatitis C Virus	Cocaine
HIV-l / HIV-2	MethadoneOpiates

1 White blood cell count included differential white blood cell count. 2 Direct microscopy was performed if the sample was positive for any of these parameters.

**Table 6:** *Experiment 1,* GET 73 Pharmacokinetics parameters: *C*_*max*_ (ng/mL), *AUC*_*0-t*_ (ng*h/mL) and *t*_*max*_ (h) by dose/treatment and day.

Dose GET 73	*C*_*max*_ (ng/mL)	*AUC*_*0-t*_ (ng*h/mL)	*t*_*max*_ (h)
10-mg	48.35 (27.47–69.23)	60.04 (25.97–94.11)	0.50 (0.5–1.0)
30-mg	309.67 (211.46–407.88)	387.36 (224.72–550.01)	0.50 (0.50–1.02)
100-mg	721.33 (250.65–1192.01)	1246.80 (690.63–1802.98)	0.75 (0.50–1.50)
300-mg	1384.67 (510.79–2258.54)	3624.01 (1451.26–5796.77)	0.75 (0.50–1.50)
450-mg	3891.67 (2442.82–5340.52)	6931.90 (3314.19–10,549.62)	0.50 (0.50–1.00)
600-mg	5015.0 (3061.38–6968.62)	13,338.25 (7495.02–19,181.48)	0.75 (0.50–1.50)

Results reported as *M* and lower to upper 95% (*CI*) and for *t*_*max*_ as median (min-max values).

**Table 7:** *Experiment 2,* GET 73 Pharmacokinetics parameters: *C*_*max*_ (ng/mL) by dose/treatment and day.

Dose GET73	Day 1 - single dose (ng/mL)	Day 14 - repeated doses (ng/mL)
100-mg	368.17 (218.85–517.48)	302.67 (151.32–454.02)
300-mg	2660.33 (1348.94–3971.73)	2075.67 (1016.19–3135.14)
450-mg	3723.33 (2774.58–4672.08)	3161.83 (2089.28–4234.39)
450-mg twice day	3271.67 (2140.81–4402.52)	4055.00 (2587.33–5522.67)

Results reported as *M* and lower to upper 95% (*CI*).

**Table 8:** *Experiment 2,* GET 73 Pharmacokinetics parameters: *t*_*max*_ (h) by dose/treatment and day.

Dose GET73	Day 1 - single dose (h)	Day 14 - repeated doses (h)
100-mg	1.00 (0.50–1.50)	1.00 (0.50–1.50)
300-mg	0.50 (0.50–1.00)	0.75 (0.50–1.50)
450-mg	1.00 (0.50–1.53)	1.00 (0.50–1.50)
450-mg twice day	0.75 (0.5–2.05)	0.75 (0.50–1.50)

Results reported as *Median* and minimum and maximum.

**Table 9:** *Experiment 2,* GET 73 Pharmacokinetics parameters: *AUC*_*0-t*_ (ng*h/mL) by dose/treatment and day.

Dose GET73	Day 1 - single dose (ng*h/mL)	Day 14 - repeated doses (ng*h/mL)	Ratio day 14/day1
100-mg	657.3 (382.67–931.59)	598.75 (369.52–827.98)	0.91
300-mg	6348.74 (2297.78–10,399.70)	5938.89 (2193.79–9684.00)	0.94
450-mg	8541.24 (6244.42–10,838.07)	8102.90 (4724.25–11,481.55)	0.95
450-mg twice day	8639.29 (5811.59–11,467.00)	9704.90 (6053.12–13,356.68)	1.12

Results reported as *M* and lower to upper 95% (*CI*).

**Table 10:** *Experiment 2,* GET 73 Pharmacokinetics parameters: *C*_*min*_ (ng/mL) by dose/treatment and day.

Dose GET73	Day 1 - single dose (ng/mL)	Day 14 - repeated doses (ng/mL)
100-mg	0 (0–0)	0 (0–0)
300-mg	0.36 (−0.35 to 1.07)	0 (0–0)
450-mg	0.36 (−0.35 to 1.07)	0 (0–0)
450-mg twice day	49.99 (−5.45 to 105.42)	39.36(12.16–66.57)

Results reported as *M* and lower to upper 95% (*CI*).

**Table 11:** *Experiment 2,* MET 2 parameters: *C*_*max*_ (ng/mL) by dose/treatment and day.

Dose GET73	Day 1 -single dose (ng/mL)	Day 14 - repeated doses (ng/mL)
100-mg	1728.33 (1415.98–2040.68)	1491.33 (1042.06–1940.61)
300-mg	4193.33 (3550.43–4836.23)	4511.67 (3828.91–5194.42)
450-mg	6691.67 (5304.95–8078.38)	6003.33 (5664.54–6342.12)
450-mg twice day	7406.67 (6299.63–8513.71)	8820.00 (7482.47–10,157.53)

Results reported as *M* and lower to upper 95% (*CI*).

**Table 12:** *Experiment 2,* MET 2 parameters: *t*_*max*_ (h) by dose/treatment and day.

Dose GET73	Day 1 - single dose (h)	Day 14 - repeated doses (h)
100-mg	1.00 (0.50–1.50)	1.00 (0.50–1.50)
300-mg	1.00 (0.50–1.50)	1.50 (1.00–2.00)
450-mg	1.25 (1.00–2.00)	1.25 (1.00–1.50)
450-mg twice day	1.25 (1.05–2.05)	1.29 (1.00–1.60)

Results reported as *Median* and minimum and maximum.

**Table 13:** *Experiment 2,* MET 2 parameters: *AUC*_*0-t*_ (ng*h/mL) by dose/treatment and day.

Dose GET73	Day 1 - single dose (ng*h/mL)	Day 14 - repeated doses (ng*h/mL)	Ratio day 14/day1
100-mg	3934.30 (3373.35–4495.25)	3790.54 (3086.52 - 4494.56)	0.96
300-mg	15,235.13 (11,392.92–19,077.34)	15,312.29 (11,615.23 - 19,009.35)	1.00
450-mg	21,051.64 (18,233.17–23,870.10)	20,693.28 (19,477.42 - 21,909.14)	0.98
450-mg twice day	30,443.09 (24,553.06–36,333.12)	32,251.41 (26,029.38 - 38,473.43)	1.06

Results reported as *M* and lower to upper 95% (*CI*).

**Table 14:** *Experiment 2,* MET 2 Pharmacokinetic parameters: *C*_*min*_ (ng/mL) by dose/treatment and day.

Dose GET73	Day 1 - single dose (ng/mL)	Day 14 - repeated doses (ng/mL)
100-mg	0 (0–0)	0 (0–0)
300-mg	0 (0–0)	0 (0 to 0)
450-mg	0 (0–0)	0 (0–0)
450-mg twice day	804.43 (−240.21 to 1849.08)	426.58 (85.56–767.71)

Results reported as *M* and lower to upper 95% (*CI*).

In Table 1, Table 2, timetables were set for the administration of GET 73 and the collection of data for the safety and tolerability of GET 73 in single and repeated ascending doses in healthy male volunteers.

In Tables 3 and 4, a procedure was documented for both single and repeated ascending doses with the goal of collecting preliminary pharmacokinetic data and monitor the safety of participants.

Table 5 lists laboratory assessments for safety of GET 73 and potential factors that can affect its tolerability. Some tests were also used for inclusion/exclusion criteria (urine tests for drugs of abuse).

Table 6 data shows the extent of exposure of GET 73 plasma concentration in a single ascending dose. *C*_*max*_ and *AUC*_*0-t*_ are measures of these factors respectively with *t*_*max*_ as a reference to the maximum concentration of GET 73 in the plasma.

Table 7 tests for *C*_*max*_ in repeated ascending dose administration with data for Day 1 and Day 14.

For Table 8, *t*_*max*_ was collected in reference to when the *C*_*max*_ was reached in the repeated ascending dose administration group for both Day 1 and Day 14.

Table 9 measures the *AUC*_*0-t*_ for repeated ascending dose administration experiment. *AUC*_*0-t*_ ratio compares Day 14 to Day 1 levels.

Table 10 shows the *C*_*min*_ of GET 73 in the plasma over the repeated ascending dose administration. Data reported for Day 1 and Day 14.

Table 11 goes into data about MET 2, the main metabolite of GET 73. Data reported for Day 1 and Day 14.

Table 12 shows the *t*_*max*_ of MET 2 in the repeated ascending dose administration of GET 73.

Data reported for Day 1 and Day 14.

Table 13 collects data on the *AUC*_*0-t*_ of MET 2 in the plasma after repeated ascending dose administration of GET 73. Ratio compares Day 14 and Day 1 data are listed as well.

Table 14 measures the *C*_*min*_ of MET 2 in the plasma for repeated ascending dose administration of GET 73. Data was collected for Day 14 and Day

## 2 Experimental design, materials and methods

The method for collection of pharmacokinetic analytical data was validated at Quotient Bioresearch Ltd, UK. The procedures for the development and the validation of the method were based on the United States (US) Food and Drug Administration (FDA) recommendations Guidance for Industry Bioanalytical Method Validation - U.S. Department of Health and Human Services, FDA Center for Drug Evaluation and Research (CDER), Center for Veterinary Medicine (CVM), May 2001, BP.

Blood samples for pharmacokinetic evaluation in plasma were collected into tubes containing lithium heparin prior to dosing and at various time points after dosing up to 48 h for *Experiment 1* and up to 360 h for *Experiment* *2*. The plasma samples were obtained from individuals from six single ascending dose groups in *Experiment 1* (10-mg, 30-mg, 100-mg, 300-mg, 450-mg and 600-mg) and four multiple ascending dose groups in *Experiment 2* (100-mg 300-mg, 450-mg and 450-mg twice a day).

Concentrations of GET 73 in human plasma samples were measured by LC–MS/MS after SLE+ (supported liquid extraction) over the calibration range of 2–1000 ng/ml according to the validated method and the relevant SOPs. Concentrations of MET 2 in human plasma samples were measured by LC–MS/MS after protein precipitation and dilution over the calibration range of 20–10,000 ng/ml. All instrument control, data collection, peak area integration and storage were performed using Analyst (versions 1.4.2 and 1.5.1, Applied Biosystems Inc., OA, US). Peak areas were then imported into Watson LIMS (version 7.2 Thermo Fisher Scientific Inc., MA, US) for regression and quantification. The mass spectrometer response (peak area ratio of analyte to internal standard) of each calibration standard was calculated by Watson LIMS and plotted against the nominal (prepared) concentration. A weighted linear regression analysis was used to calculate an equation of the calibration line. Concentrations of GET 73 and MET 2 in the plasma samples were back calculated from the calibration lines.

Values for the following pharmacokinetic parameters were estimated: 1) maximum plasma concentration (*C*_*max*_), 2) the time to reach maximum plasma concentration (*t*_*max*_) and 3) the area under the plasma concentration time curve over the dosing interval (*AUC*_*(0-t)*_). Pharmacokinetic parameters were derived by noncompartmental analysis using WinNonlin (Version 4.1b, Pharshight Corporation, Mountain View, CA, US). Data in this article has not been published elsewhere.
