# Supplementary material for: Dataset for Phase I randomized clinical trial for safety and tolerability of GET 73 in single and repeated ascending doses including preliminary pharmacokinetic parameters
Source: Data Brief. 2017 Sep 17;15:407–13. doi: 10.1016/j.dib.2017.09.018 (PMC5712048; doi:10.1016/j.dib.2017.09.018)
Supplement: Supplementary file 1 — Supplementary material [file mmc1.pdf]

AUTHORS DECLARATION

Title: Dataset for Phase I clinical trial for safety and tolerability of GET 73 in single and repeated ascending doses including preliminary pharmacokinetic parameters

Authors: Carolina L. Haass-Koffler[a,b], Kimberly Goodyear[b], Victoria M. Long[b], Harrison H. Tran[b], Antonella Loche[c], Roberto Cacciaglia[c], Robert M. Swift[a] and Lorenzo Leggio[b]

Affiliations: [a]Center for Alcohol and Addiction Studies, Department of Psychiatry and Human Behavior, Brown University, Providence, RI, USA; [b]Center for Alcohol and Addiction Studies, Department of Behavioral and Social Sciences, Brown University, Providence, RI, USA; [c]Laboratorio CT, San Remo, Italy

We wish to draw the attention of the Editor to the following facts which may be considered as potential conflicts of interest and to significant financial contributions to this work.

This study was funded by CT Laboratories, San Remo, Italy. Roberto Cacciaglia and Antonella Loche are employees of CT Laboratories. Robert M Swift has received consultant fees from CT Laboratories and travel and honoraria from D&A Pharma and Lundbeck. The other authors report no biomedical financial interests or potential conflicts of interest.

We confirm that the manuscript has been read and approved by all named authors and that there are no other persons who satisfied the criteria for authorship but are not listed. We further confirm that the order of authors listed in the manuscript has been approved by all of us.

We confirm that we have given due consideration to the protection of intellectual property associated with this work and that there are no impediments to publication, including the timing of publication, with respect to intellectual property. In so doing we confirm that we have followed the regulations of our institutions concerning intellectual property.

We further confirm that any aspect of the work covered in this manuscript that has involved human patients has been conducted with the ethical approval of all relevant bodies and that such approvals are acknowledged within the manuscript.

We understand that the Corresponding Author is the sole contact for the Editorial process (including Editorial Manager and direct communications with the office). She is responsible for communicating with the other authors about progress, submissions of revisions and final approval of proofs. We confirm

that we have provided a current, correct email address which is accessible by the Corresponding Author and which has been configured to accept email from

carolina_haass-koffler@brown.edu

Signed by all authors as follows:

Carolina L. Haass-Koffler	[illegible]
Kimberly Goodyear	[illegible]
Victoria M. Long	Victoria m Long
Harrison H. Tran	[illegible]
Antonella Loche	[illegible]
Roberto Cacciaglia	[illegible]
Robert M. Swift	[illegible]
Lorenzo Leggio	Lorenzo Leggio
